# Delayed Motor Milestones Achievement in Infancy Associates with Perturbations of Amino Acids and Lipid Metabolic Pathways

**DOI:** 10.3390/metabo10090337

**Published:** 2020-08-19

**Authors:** Rebecca Kofod Vinding, Daniela Rago, Rachel S. Kelly, Gözde Gürdeniz, Morten Arendt Rasmussen, Jakob Stokholm, Klaus Bønnelykke, Augusto A. Litonjua, Scott T. Weiss, Jessica Lasky-Su, Hans Bisgaard, Bo Lund Chawes

**Affiliations:** 1 COPSAC, Copenhagen Prospective Studies on Asthma in Childhood, Herlev and Gentofte Hospital, University of Copenhagen, 1017 Copenhagen, Denmark; rebecca.vinding@dbac.dk (R.K.V.); daniela.rago@dbac.dk (D.R.); gozde.gurdeniz@dbac.dk (G.G.); morten.arendt@dbac.dk (M.A.R.); stokholm@copsac.com (J.S.); kb@copsac.com (K.B.); chawes@copsac.com (B.L.C.); 2 Channing Division of Network Medicine, Brigham and Women’s Hospital and Harvard Medical School, Boston, MA 02115, USA; hprke@channing.harvard.edu (R.S.K.); restw@channing.harvard.edu (S.T.W.); jessica.a.su@gmail.com (J.L.-S.); 3 Department of Food Science, University of Copenhagen, 1958 Frederiksberg, Denmark; 4 Division of Pediatric Pulmonary Medicine, Golisano Children’s Hospital, University of Rochester Medical Center, Rochester, NY 14642, USA; augusto_litonjua@urmc.rochester.edu

**Keywords:** motor milestones, metabolomics, children, neurodevelopment

## Abstract

The relationship between developmental milestone achievement in infancy and later cognitive function and mental health is well established, but underlying biochemical mechanisms are poorly described. Our study aims to discover pathways connected to motor milestone achievement during infancy by using untargeted plasma metabolomic profiles from 571 six-month-old children in connection with age of motor milestones achievement (Denver Developmental Index) in the Copenhagen Prospective Studies on Asthma in Childhood 2010 (COPSAC2010) mother–child cohort. We used univariate regression models and multivariate modelling (Partial Least Squares Discriminant Analysis: PLS-DA) to examine the associations and the VDAART (Vitamin D Antenatal Asthma Reduction Trial) cohort for validation. The univariate analyses showed 62 metabolites associated with gross-motor milestone achievement (*p* < 0.05) as well as the PLS-DA significantly differentiated between slow and fast milestone achievers (AUC = 0.87, *p* = 0.01). Higher levels of tyramine-O-sulfate in the tyrosine pathway were found in the late achievers in COPSAC (*p* = 0.0002) and in VDAART (*p* = 0.02). Furthermore, we observed that slow achievers were characterized by higher levels of fatty acids and products of fatty acids metabolism including acyl carnitines. Finally, we also observed changes in the lysine, histidine, glutamate, creatine and tryptophan pathways. Observing these metabolic changes in relation to gross-motor milestones in the first year of life, may be of importance for later cognitive function and mental health.

## 1. Introduction

The brain is the most complex organ of the body and undergoes developmental changes throughout life, where the earliest phases of maturation during fetal development and childhood are the most important for later mental health [1]. Skills, such as smiling, grasping, crawling, the first walking steps, and the first spoken word, are known as developmental milestones and are divided into gross-motor, fine motor, language, and social skills. During infancy there is a rapid increase in motor abilities: the child learns to reach, grasp, sit, stand and walk. These motor abilities or milestones in early life are universal and the WHO has classified the age ranges at which these developmental milestones are normally achieved [2].

Delayed achievements of motor milestones are known prodromal events of mental impairments, including intellectual disabilities [3], and even minor deviations can predict abnormal cognition and language development [4,5,6]. Some predictors of late attainment of motor milestones are gestational age, low birth weight, short period of breastfeeding, reduced height and weight, small head size increase in the first year of life [7], and insufficient nutrient intake [8]. Early attainment is associated with better cognitive outcomes in adulthood, such as higher education, better executive function, and a higher level of intelligence [9]. Furthermore, it has been shown that delayed motor development associates with psychopathological disorders such as schizophrenia, autism and neurosis [7,10].

**Figure 1:**
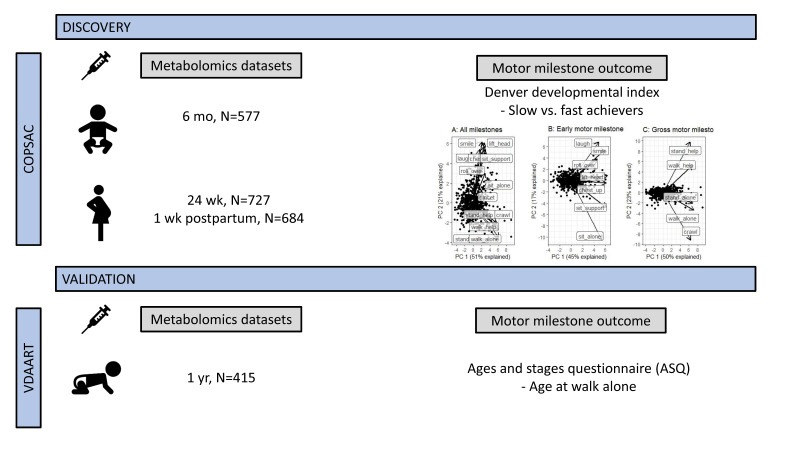
Experimental design of the study.

Even though the relationship between milestone achievement and later mental health is well established, the biochemical pathways affecting age of motor milestone achievement are still poorly understood. This exploratory study based on untargeted blood metabolomic profiles from young children aims to discover possible biomarkers and pathways connected to motor milestone achievement during infancy. For discovery, we investigated the association between age six months blood metabolomic profiles and age at motor milestone achievement following the Denver Developmental Index among children participating in the Copenhagen Prospective Studies on Asthma in Childhood 2010 (COPSAC2010) mother–child cohort. Thereafter, we investigated whether the metabolic signature of age at motor milestone achievement in the child was dependent on the maternal metabolic profile during pregnancy; i.e., gestational week 24 and 1 week postpartum. Finally, we validated our findings by exploring the relationship between metabolomic profiles at age 1 year and age at walking alone in the independent Vitamin D Antenatal Asthma Reduction Trial (VDAART) birth cohort (Figure 1). To our knowledge this is the first metabolomics study of these developmental phenotypes.

## 2. Results

### 2.1. Baseline Characteristics

A total of 571 (82%) of the 700 children in the COPSAC2010 cohort had complete data on both milestones and plasma metabolomic profiling at six months of age. Mothers of the children, who were excluded based on incomplete data were more likely to be nulliparous, to have antibiotic treatment during pregnancy and were slightly older when giving birth. The excluded children had a lower gestational age due to the exclusion of children born premature and had a shorter duration of solely breastfeeding (Table S1).

### 2.2. Gross-Motor Milestones

The association analyses between the child 6mo (six months) metabolic profiles and gross-motor milestone PC1, identified 62 metabolites as nominally significant among the 984 metabolites investigated, with 10 out of 62 being positively associated, including tyramine-O-sulfate as the strongest finding (β-estimate 0.30, CI 0.14–0.46, SE 0.08). The predominance of negative associations may partially be explained by metabolite correlations, i.e., data collinearity (see correlation map, Figure S1).

**Figure 2:**
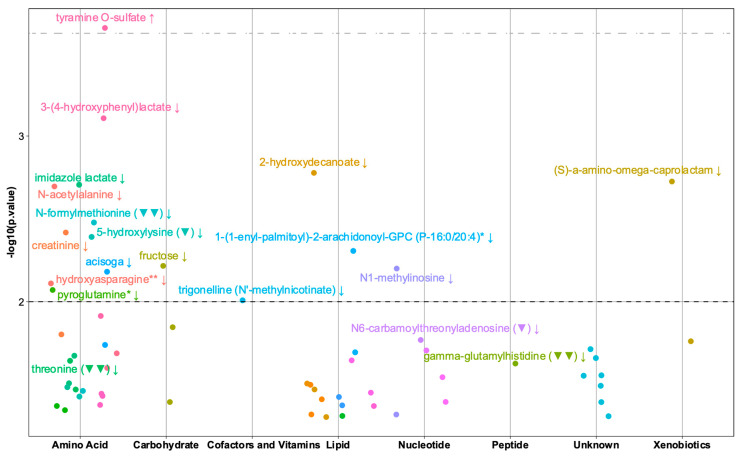
Plot of the metabolites in the children at six months significantly associated with gross-motor milestone achievement. Each dot represents a metabolite grouped by super-pathway (x-axis) with associated −log10 (*p*-value) on the y-axis. The black dotted line represents a 0.01 *p*-value cut-off, whereas the grey dotted line represents a 0.25 *q*-value cut-off. The labelled dots are metabolites having *p*-value ≤ 0.01 or being also associated in the mother metabolome. The arrows represent the direction of the association with the gross-motor milestone. Metabolites in each super-pathway having the same color are from the same sub-pathway. (▼): A significant association (*p* ≤ 0.05) between the mother metabolite level both at w1 and child gross-motor milestone. The direction of the association is as in the child metabolome. (▼▼): A significant association (*p* ≤ 0.05) between the mother metabolite level both at w1 and w24 and child gross-motor milestone. The direction of the association is as in the child metabolome.

To disentangle whether the observed metabolic profile in the child 6mo samples was related to the maternal metabolic profile, we investigated this early metabolic signature in the mother’s w24 and w1 metabolic profiles. Considering the nature of the investigation being exploratory and the collinearity of the metabolomics data, we included all the nominally significant metabolites in the analysis. The results from the child 6mo and mother w24 and w1 metabolomics data are presented in Table S2 and a summary of the main results is visualized in Figure 2.

The amino acids group is over-represented in the association analyses with the tyrosine pathway being the most perturbated with increased tyramine O-sulfate and decreased 3-4-hydroxyphenyllactate, followed by decreased levels of several metabolites including imidazole lactate (histidine pathway), 5-hydroxylysine, N-acetylalanine, N-formylmethionine (conjugated methionine) and creatinine. The latter might be connected with creatine, which is also associated with gross-motor milestone achievement, but less significantly than tyrosine.

When considering the association between mother metabolite levels at both time points vs. child gross-motor milestones achievement, we observed that three metabolites out of 62, N-formylmethionine, threonine (essential amino acids), and gamma-glutamyl-histidine (histidine containing peptide) were significant and showed the same direction. Two more metabolites from the mother w1 profile were significantly associated with gross-motor milestones, N6-carbamoylthreonyladenosine (purine metabolism) and 5-hydroxylysine. To assess independency from mother levels in the 6mo analyses, we made a correlation analysis based on the Spearman correlation between mother w24 and w1 and child 6mo for those five metabolites, showing that the most correlated metabolites between mother and child were N-formylmethionine and 5-hydroxylysine (Figure S2). Adjusting the child 6mo models for mother w24 and w1 levels for these five metabolites and the tyramine O-sulfate (as main finding) showed similar results, i.e., the results were independent from the mother’s levels (Table S3).

**Figure 3:**
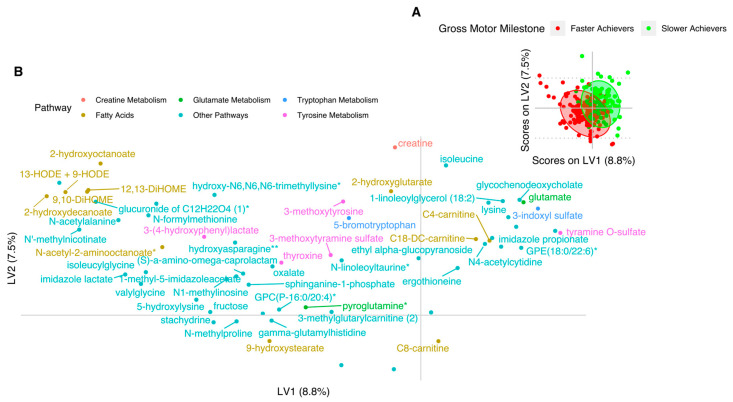
Partial Least Squares Discriminant Analysis (PLS-DA) model of the children’s plasma metabolome in relation to the gross-motor milestone achievement. (**A**) Scores plot; (**B**) Loadings plot.

Subsequently, we applied a Partial Least Squares Discriminant Analysis (PLS-DA) multivariate analysis to investigate possible patterns of metabolites associated with slow vs. fast gross-motor milestone achievement; i.e., children belonging to the first quartile (N = 132) vs. last quartile (N = 132) of PC1 in the gross-motor milestone Principal Component Analysis (PCA). The model validation is presented in Figure S3 showing that the average of the non-permuted models performed significantly better than the permuted. The non-permuted 100 models were used to build a final model, which consisted of two components and 62 metabolites (see Figure 3 and Table S4). This final model had an Area Under the Curve AUCcv = 0.87, Classification Error CEcv = 0.21 and a *p*-value = 0.005 (based on 100× permutations).

From the scores plot in Figure 3, we observed that tyramine-O-sulfate (tyrosine pathway), glutamic acid (glutamate metabolism), glycochenodeoxycholate (primary bile acid), lysine and isoleucine in LV1 as well as creatine in LV2 discriminated the late-achieving children. Acetyl-alanine, N-methylnicotinate (nicotinate metabolism), imidazole lactate, 3-4-hydroxyphenyllactate, gamma-glutamylhistidine, valylglycine (dipeptide), 5-hydroxylysine, some of the Hydroxyoctadecadienoic acid (HODE) and Dihydroxy-9Zoctadecenoic acid (DiHOME) metabolites together with 2-hydroxyoctanoic and 2-hydroxydecanoic acids (all part of the fatty acid family) discriminated fast-achieving children. Thus, the multivariate approach confirmed some of the strongest univariate findings, but also broadened the picture with patterns of metabolites, taking into account some of the collinearity in the data.

### 2.3. Validation in VDAART

To validate our findings and at the same time take into account the challenge in doing so, both in terms of differences in motor milestone outcomes between the two cohorts, different metabolomics profiling age-point, i.e., six months vs. one year, and the uncertainty of the univariate findings, we only tested the strongest associated metabolite, tyramine-O-sulfate which passed the False Discovery Rate (FDR) cut-off for the validation. This metabolite was significantly associated with an earlier age of walking alone in the VDAART cohort (β-estimate 0.89, *p* = 0.02), which replicated our findings in COPSAC. Furthermore, we also tested the association of tyramine-O-sulfate levels with age of walking alone as an individual milestone to align with the VDAART milestone showing similar results (β-estimate 10.71, *p* = 7.10 × 10^−5^).

### 2.4. Early Motor Milestones

The association analyses between the child 6mo metabolic profiles and early motor milestone achievement resulted in 89 nominally significant metabolites, but none of the metabolites had a *q*-value ≤ 0.25. Furthermore, we were unable to validate the multivariate PLS-DA model for predicting the early motor milestone outcome (area under the receiver operator characteristic curve: AUROC 0.52, CE 0.48).

**Figure 4:**
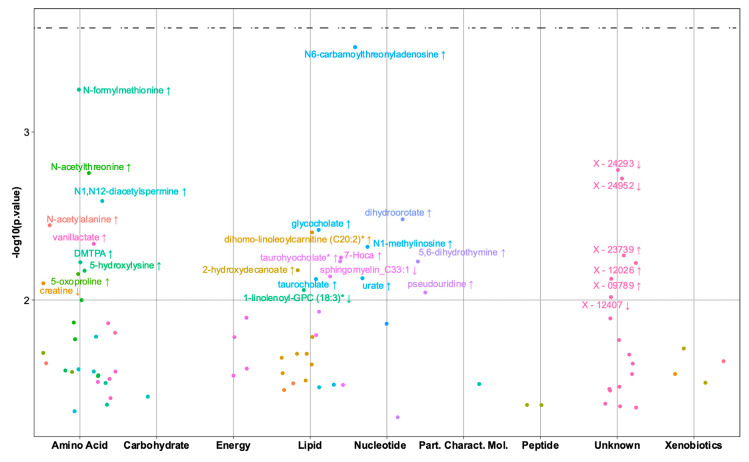
Metabolites in the children at age six months significantly associated with early motor milestones achievement. Each dot represents a metabolite grouped by super-pathway (x-axis) with associated −log10 (*p*-value) in the y-axis. The grey dotted line represents a 0.01 *p*-value cut-off, whereas the black dotted line represents a 0.25 *q*-value cut-off. The labelled dots are the metabolites having *p*-value ≤ 0.01. The arrow symbol indicates the direction of the association.

All the metabolites with *p* ≤ 0.05 in the univariate analyses are reported in Table S5, and metabolites with *p* ≤ 0.01 are shown in Figure 4. Contrary to association with the gross-motor milestones, the associations are mainly positive; however, the metabolites are scattered over different pathways. Among the metabolites with *p* ≤ 0.01, N-formylmethionine, 5-hydroxylysine, creatine, N-acetylalanine and primary bile acids were associated with both gross-motor and early motor milestones, but with the exception of creatine, in the opposite direction.

## 3. Discussion

In this first exploratory study examining metabolites and biochemical pathways underlying the achievement of motor milestones in early life, we observed that the achievement of gross-motor milestones was associated with perturbations in different classes of amino acids and lipids by six months of age in a large population-based birth cohort of Danish children. In particular, we observed perturbations in the tyrosine pathway, which is known to lead to production of the catecholamine neurotransmitters, which were replicated in an independent American birth cohort. These novel findings gain insight into the metabolic regulation of motor development in childhood, which may be of importance for the risk of psychiatric diseases later in life.

**Figure 5:**
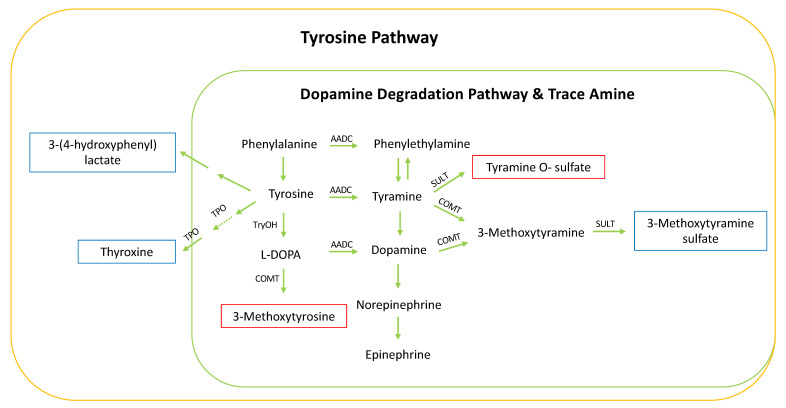
Metabolites in the tyrosine pathway associated with gross-motor milestones achievement. The metabolites present in the multivariate model are reported in a frame color based on the association with the milestone: the fast achievers (blue) or slow achievers (red).

Achievement of gross-motor milestones was associated with metabolites in the tyrosine pathway in both the COPSAC and VDAART cohorts, including an increased level of tyramine O-sulfate and 3-methoxytyrosine and a decreased level of 3-methoxytyramine sulfate, 3-(4-hydroxyphenyl) lactate and thyroxine in slow achievers (see pathway map in Figure 5).

Tyramine conjugated metabolites are degradation products of tyramine, a trace amine originating from the decarboxylation of tyrosine, whereas 3-methoxytyrosine is a degradation product from levodopa. Tyramine is present in the brain and in the blood, but at low concentration compared to catecholamines since it has a very rapid turnover and is not stored in the body [11]. Tyramine releases norepinephrine that has a vasoconstriction effect and increases cardiac output [12]. We observed that levels of tyramine O-sulfate and 3-methoxytyrosine were higher in children who were slow achievers of gross-motor milestones, whereas 3-methoxytyramine sulfate was lower compared to children who were fast achievers. A possible explanation might be that among slow achievers there is less dopamine produced or available from tyramine or L-DOPA, which could also explain the lower levels of 3-methoxytyramine sulfate that is a conjugated product from dopamine catabolism. We also observed that fast achievers compared to slow achievers had a higher level of 3-(4-hydroxyphenyl) lactate, which is a metabolite that can also be produced by the gut microbiota and has been associated with the suppression of reactive oxygen species in mitochondria and neutrophils [13]. Finally, we observed that fast achievers had a higher level of thyroxine, which is probably an exercise response [14]. Thus, our observation of perturbations in the tyrosine pathway could be directly related to the activity level of the child. However, previous studies have shown that delayed attainment of milestones in infancy is associated with the risk of developing schizophrenia [15] and growing evidence suggests that dopamine dysregulation plays a role in the pathogenesis of schizophrenia [16,17]. For mother w24 and w1 metabolic profiles, none of the compounds from the tyrosine pathway were associated with gross-motor milestones achievement. This indicates that the modifications within the tyrosine pathway reflecting gross-motor milestones were either not transmitted from the mothers, or that the stronger variation due to lifestyle factors in the mothers such as diet and postprandial sampling is masking the possible effects.

Creatine, a guanidine compound which can be synthetized in the liver or acquired through diet, appeared to be an important metabolite for gross-motor milestone achievement in the multivariate model. The de novo creatine is produced from the methylation of guanidino acetate at the amidino group and is then transported via the blood to the muscles, where most of it is found. Creatine is successively transformed to phosphoryl-creatine, which is a high-energy compound used during energy demand for regenerating ATP from ADP. However, phosphoryl-creatine and creatine are also non-enzymatically partially converted in a steady state to creatinine, which is then excreted into the urine [18]. Creatinine is proportional to the muscle mass and its urine and serum levels increase due to physical activity [19]. We observed an increased level of creatine and at the same time a decreased level of creatinine in children who were slow achievers of gross-motor milestones, which is presumably a result of being less active.

Slow milestone achieving children were also characterized by a higher level of lysine and lower level of 5-hydroxylysine, which are components of collagen tissue. Lysine is a marker of collagen synthesis [20] and 5-hydroxylysine of collagen breakdown as it originates from the hydroxylation of lysine in the alpha-chain of collagen. The hydroxylysine residues are important for stabilizing the fiber forming cross-links and are anchor points for carbohydrate attachment [21]. The compound is found in the urine as a free, or more often conjugated form, such as galactosyl-5-hydroxylysine or glucosyl-galactosyl-5-hydroxylysine [20,21,22]. The latter forms are useful for assessing resorption of bone collagen and are not affected by the diet [23]. The free form of 5-hydroxylysine accounts only for 10% of the total excretion and it is not known if it comes directly from the collagen or from the conjugated forms [22]. Children normally have high collagen turnover [24], and in our study it seemed that fast achievers had lower lysine and higher 5-hydroxylysine levels compared to slow achievers, probably reflecting a perturbated collagen metabolism due to their more active state. Interestingly, the maternal 5-hydroxylysine level at w1 was also higher among fast achieving children suggesting that a maternal lifestyle of being more active influences the child’s gross-motor milestone achievement. 

More active children who achieve motor milestones faster may have higher exercise-induced oxidative stress compared to less active children [25]. This is supported by our observation that fast achieving children had higher levels of gamma-glutamyl histidine (a gamma-glutamyl amino acid) presumably resulting from an increased activity of the gamma-glutamyl transferase as a response to increased synthesis of reduced glutathione in skeletal muscles [25].

Furthermore, we observed higher levels of medium chain alpha-hydroxy fatty acids (alpha-hFA), including 2-hydroxydecanoic acid, 12,13-DiHOME, 9,10-DiHOME, and 13+9-HODE in children who were fast compared to slow achievers of gross-motor milestones. The latter metabolites are lipokines, i.e., lipid hormones formed in the adipose tissue, which regulate systemic homeostasis [26]. They are P450 cytocrome-derived linoleic acid metabolites, and an increased level of circulating 12,13-DiHOME from brown adipose tissue has been observed in response to exercise and linked to an increased fatty acid uptake in the skeletal muscle [27]. These compounds and other lipokines are therefore plausible markers of activity. However, alpha-hFA formed by hydroxylation of Fatty acid (FA) by the fatty acid hydroxylase 2 (FAH2) enzyme can be incorporated into hFA-ceramides and the more complex hFA-sphingolipids [28], which have the highest concentration in brain myelin sheaths. FAH2 enzyme deficiency in the brain leading to lower formation of hFA-sphingolipids is associated with brain disorders such as leukodystrophy [29,30,31,32,33].

Finally, we observed that slow achieving children had higher levels of imidazole propionate and lower imidazole lactate. Both compounds are histidine-derived metabolites, which can also be produced by the gut microbiota [34,35] and may therefore indicate a possible difference in the gut microbiome composition between children who are fast compared to slow achievers of gross-motor milestones.

To our knowledge, this is the first exploratory study utilizing metabolomics to investigate metabolites and biochemical pathways associated with the achievement of motor milestones in early life among healthy children. This field of research is new and we believe that our findings are of importance in the light of the associations found between early neurodevelopment, including achievement of motor milestones, and the risk of psychiatric diseases, intelligence and executive function in adult life [36,37]. Interestingly, early achievement of gross-motor milestones has been associated with better language development in subpopulations of patients with autism spectrum disorders [38]. Previous studies have shown dysregulation in serotonin metabolites from the tryptophan pathway in patients with autism compared to controls [39], but perturbations in levels of fatty acids, sterols, phospholipids, and molecules associated with oxidative stress have also been proposed as potential biomarkers for autism [40,41]. This, together with studies showing dopamine dysregulation in patients with schizophrenia [16,17] supports a role for metabolomics in psychiatry [42] and our findings related to motor milestone achievement in early life may be important for predicting the risk of mental health later in life. It is significant strength of our study that COPSAC is an unselected population-based cohort with a longitudinal design, closely following the children from birth throughout infancy with prospective collection of age at milestone achievement along with a large amount of environmental exposures, which we used to adjust the models from potential confounders. Further, we were able to replicate our main finding in VDAART, a comparable independent cohort, despite the fact that the metabolomic profile in VDAART was from the age of one year and the gross-motor milestone was a cross-sectional score of the ability to walk alone at one year of age.

It is a limitation that our profiles are not from fasting samples and that we could not adjust our models for intake of food substances in the hours before the blood sample. However, we adjusted for information on solely breastfeeding and most children at six months of age are introduced to a limited amount of foods. Furthermore, the multiple testing correction was also another limitation in our study, however we handled it through validation of our main finding in an independent cohort.

Future studies should focus on having several time points for blood sampling during infancy and assure that the blood samples are collected after fasting. If it could be possible to determine disturbance in several metabolic pathways early in life, which associate to delay in motor development or later psychiatric disease, this could work as a tool for early detection and possibly, interventions.

## 4. Materials and Methods

### 4.1. Study Population

The Copenhagen Prospective Studies on Asthma in Childhood 2010 (COPSAC2010) is a cohort of 738 unselected pregnant women and their 700 children followed prospectively from birth until age 10 years [43]. During third trimester of pregnancy the women participated in a factorial-designed, double-blind, randomized controlled trial (RCT) of high-dose vitamin D (2400 IU/day) or standard dose (400 IU/day) and either 2.4 g n−3 long-chained polyunsaturated fatty acid (LCPUFA) or placebo capsules. Exclusion criteria were maternal chronic diseases other than asthma or a vitamin D intake above 600 IU/day. Children with a gestational age < 32 weeks were excluded.

### 4.2. Ethics

The study was conducted in accordance with the principles of the Declaration of Helsinki and was approved by the Local Ethics Committee (H-B-2008-093) and the Danish Data Protection Agency (2015-41-3696). Both parents gave written informed consent before enrolment.

### 4.3. Developmental Milestones

At the visit to the COPSAC research clinic one week postpartum the parents received a registration form with thorough instructions, based on The Denver Development Index and WHO milestones registration. Dates of achievement of 13 predefined milestones were registered prospectively by the parents and reviewed at each visit to the research clinic. The registration form contained a description and an illustration of the developmental milestones. Any difficulties in remembering the specific date were registered as “missing”. The clinical staff carefully reviewed the forms with the parents in order to standardize the registrations and minimize differences in interpersonal interpretations. Implementation of milestone registration started after the first 500 children were born and therefore some of the milestones were registered retrospectively [44].

### 4.4. Metabolomic Profiling

Blood samples for metabolomic profiling were collected from 602 children from age six months to two years. Of those, only samples from children aged 4–8 months were included in the analysis, termed child age six months (6mo) [45]. Untargeted plasma metabolomic analysis was carried out by Metabolon, Inc. (Morrisville, NC, USA) using 4 platforms: (1) UPLC-ESI(+)MS/MS optimized for hydrophilic compounds; (2) UPLC-ESI(+)MS/MS optimized for hydrophobic compounds; (3) reverse phase UPLC-(-)MS/MS using basic optimized conditions, and (4) HILIC/UPLC-(-)MS/MS. Details on sample preparation, LC-MS/MS analysis and quality control are detailed previously [45]. The metabolomic profiles contained 1138 unique metabolites that were identified based on three matching criteria: retention time/index (RI) range, mass accuracy (±10 ppm) and MS/MS spectra. The compound identification was based on the following criteria: (1) compounds labelled with “*” have identification level 2; (2) compounds labelled with “**” have level 3 (since no standards or matching spectra are available); (3) compounds named with “X-” are unknown and therefore have level 4, and (4) if no label is applied, the identification level is 1 [46].

Blood samples were also collected from the mothers at week 24 of pregnancy (w24) and one week postpartum (w1) and plasma metabolomic profiles were generated following the same procedure as used for the child 6mo samples.

### 4.5. Covariates

Information on race, gender, gestational age, maternal age at birth, household income, parent’s educational level and paternity leave were obtained by parental interviews and if possible validated with register data. Information about duration of exclusive and total breastfeeding length was obtained during scheduled visits to the research clinic and recorded online in a dedicated database. The social circumstances in the household were defined as the first component of a PCA on household income, maternal age and maternal level of education, explaining 55% of the variance [47]. Anthropometric measurements were done in the COPSAC clinic and WHO age and gender specific BMI z-scores were calculated [2].

### 4.6. Statistical Analysis

#### 4.6.1. Data Pre-Processing

Metabolomics data: To compensate for the inter-day variation, each compound was corrected in run-day blocks by registering the medians to equal one and normalizing each variable, accordingly. The final set of variables from all four metabolomic platforms was imported into Matlab (Version 9.3, the Mathworks, Inc., Natick, MA, USA) and R (Version 3.6.0, Boston, MA, USA) for statistical analysis. To remove spurious information prior to data analysis, samples with ≥30% of missing values and compounds containing ≥95% of missing values were discarded, leaving 984 metabolites. Finally, missing values were imputed with zeros.

Milestones: The developmental milestones have previously been combined in a PCA model, where all the milestones are represented in the first principal component (PC1) explaining 51% of the variance (Figure S4A) [44]. PC2 explained 21% variance and subdivided the milestones into gross-motor milestones (crawling, walking, standing) and early milestones (smiling, laughing, lifting the head, rolling and sitting with support). Due to the fact that those two groups of milestones represent different categories of motor achievements, we subsequently treated them as separated groups and built two new PCA models. In both models, PC1 is the component explaining most of the variance and is therefore used for association with the metabolome (Figure S4B,C). The higher the PC1 score, the younger the age at milestone achievement for both PCA models.

#### 4.6.2. Univariate Analysis

Univariate linear regression analyses were performed to compare the abundance of each plasma metabolite present in the child at 6mo and in the mother at w24 and w1 with the gross-motor and early milestones achievement. All analyses were adjusted for solely breastfeeding, gender, the Long Chain Polyunsaturated Fatty Acid (LCPUFA) and vitamin D RCT treatment groups, maternal pre-pregnancy BMI, social circumstances and maternal age. Furthermore, when using the child metabolome data, child BMI and head circumference at the time of blood sampling were also used.

Metabolites associated with motor milestone achievement in the child 6mo dataset at a nominal significance cut-off (*p*-value ≤ 0.05) were also tested for association with milestones using the mother w24 and w1 datasets to investigate whether the findings were also apparent for maternal levels. Finally, we made a post analysis adjusting the significant child 6mo models for maternal levels.

Correlation Spearman analysis was used to assess metabolites–metabolite correlations and the “pheatmap” package (R Version 3.6.0, Boston, MA, USA) to draw the heatmap.

#### 4.6.3. Partial Least Squares Discriminant Analysis (PLS-DA)

To explore the association between childrens’ metabolite levels and motor milestone achievement in a multivariate setting we employed supervised PLS-DA models. The milestone PC1 expressed as a continuous variable was split into quartiles and only the samples in the lower quartile and the upper quartile expressed as a binary class were utilized for the analysis.

The metabolites were auto-scaled and the PLS-DA was performed using the PLS_Toolbox (Version 8.61, Eigenvector Research, Inc., Boston, MA, USA). The dataset was randomly split into a training set and validation set using 10% of the data but assuring that each class was equally represented in the validation test set. A 10-fold cross-validation was employed in the training set to calculate the optimal number of components based on the lowest misclassification error and 10% of the lowest variable importance for projection (VIP). Metabolites were iteratively excluded until the model reached the best performance in terms of classification error (CE). The predictive performance of the model based on the validation set was assessed using the classification error and the area under the receiver operator characteristic curve (AUROC). This procedure was repeated 100×. The global predictive performance from all the 100 models was defined as the mean of the 100× CE and 100× AUROC values. Successively, this procedure was repeated 100× using a random permuted class. The distribution of the 100 averaged AUROC and CE was compared against the averaged AUROC and CE of the non-permuted models.

The final set of metabolites was chosen based on the common variables present in at least 90% of the models and used to build a final PLS-DA model. Furthermore, a permutation test using 100× iterations was applied to assess the classification performance.

#### 4.6.4. Validation of Findings

The Vitamin D Antenatal Asthma Reduction Trial (VDAART) mother-child cohort [48] was used to validate the univariate metabolite findings passing a *q*-value ≤ 0.25 for child 6mo in relation to gross-motor milestones, using VDAART child age one year plasma metabolomic profiles, which were also acquired by Metabolon Inc. using the same analytical platforms as in COPSAC.

The motor milestone in the VDAART cohort was expressed as a continuous score of the ability to walk alone. The score was generated from the age 1 year ages and stages questionnaire (ASQ) containing three questions on the ability to walk alone. Each answer had an associated score: 0 for “yes”, 1 for “sometimes”, and 2 for “not yet”, from which the final continuous score was generated. The univariate analyses were adjusted for gender, breastfeeding, vitamin D supplementation, maternal pre-pregnancy BMI, maternal age, marital status, race and study site.

## 5. Conclusions

In this exploratory metabolomic study, we show that late achievement of gross-motor milestones such as age at standing and walking alone is associated with perturbations of amino acids and lipids in the first year of life, which may be of importance for later cognitive function and risk of psychiatric diseases.

## Supplementary Materials

The following are available online at https://www.mdpi.com/2218-1989/10/9/337/s1, Figure S1: Correlation map of the nominal significant metabolites in the association between child 6 months metabolome and gross motor milestone, Figure S2: Correlation map between the significant metabolite level in the child at 6 months and the mother at week 24 and week 1, Figure S3: Results from the PLS-DA model validation, Figure S4: Biplot from principal component analysis of all the 13 milestones. A: Biplot from principal component analysis of all the 13 milestones. Principal component 1 and 2 (PC1 and PC2) explain 51% and 21% of the overall variation in the data, respectively. B: Biplot from principal component analysis of the 7 early motor milestones. Principal component 1 and 2 (PC1 and PC2) explain 45% and 17% of the overall variation in the data, respectively. C: Biplot from principal component analysis of the 5 gross motor milestones. Principal component 1 and 2 (PC1 and PC2) explain 50% and 23% of the overall variation in the data, respectively. Table S1: Baseline characteristics of the 700 children in the COPSAC2010 cohort, Table S2: Nominal significant association (*p*-value ≤ 0.05) between metabolites level in children at 6 months of age and in mothers at week 24 of pregnancy and at week 1 after birth and gross-motor milestone achievement, Table S3: Association between metabolites level in children at 6 months of age and gross-motor milestone achievement. Analysis adjusted for mother’s metabolite level at week 24 and week 1, Table S4: Metabolites in the PLS-DA model and their associated VIP value, Table S5: Nominal significant association (*p*-value ≤ 0.05) between metabolites level in children at 6 months of age and early motor milestones.
