# Supplementary material for: Delayed Motor Milestones Achievement in Infancy Associates with Perturbations of Amino Acids and Lipid Metabolic Pathways
Source: Metabolites. 2020 Aug 19;10(9):337. doi: 10.3390/metabo10090337 (PMC7570268; doi:10.3390/metabo10090337)

Supplementary figures and tables:

Table S1. Baseline characteristics of the 700 children in the COPSAC2010 cohort.

	**Study Group N = 571**	**Drop-out N = 129**	***P*-value**
Demographics			
Caucasian% (N)	96 (548)	94,5(122)	0.64
Male % (N)	51 (291)	53.3 (69)	0.67
Mother age at birth-mean (SD)	32.01 (4.45)	32.88 (4.76)	0.05
Social circumstances mean (SD)	−0.02 (1.01)	0.07 (0.96)	0.37
**Pregnancy**			
Nulliparity % (N)	59 (45.7)	318 (55.7)	0.05
Season of birth			0.32
Winter % (N)	32.2 (184)	38.0(49)	
Spring % (N)	20.8 (119)	24.0 (31)	
Summer % (N)	23.6 (135)	20.2(26)	
Fall % (N)	23.3 (133)	17.8 (23)	
Cat or dog in pregnancy % (N)	36.1 (206)	31.0 (40)	0.32
Antibiotics in pregnancy % (N)	32.5 (185)	44.2 (57)	0.03
Pre-eclampsia % (N)	4.6 (26)	4.7 (6)	0.89
Delivery method			0.46
Vaginal % (N)	79.3 (453)	74.4 (96)	
Planned caesarean section % (N)	9.1 (52)	10.9 (14)	
Acute cesarean section % (N)	11.6 (66)	14.7 (19)	
Maternal pre-pregnancy BMI Mean (SD)	24.66 (4.53)	24.02 (3.68)	0.14
Gestational age, mean (SD)	279.89 (10.53)	275.06 (15.08)	0.001
Age of solely breastfeeding	105.54 (58.29)	92.15 (66.26)	0.02
Maternal asthma; % (N)	26.4 (151)	27.9 (36)	0.89
Smoking during pregnancy; % (N)	6.8 (39)	10.1(13)	0.28
**Anthropometrics**			
Head circumference at 6 months; Mean (SD)	43.81 (1.34)	43.72 (1.48)	0.49
z-score BMI at 6 months; mean (SD)	0.04 (0.91)	0.23 (0.98)	0.04

Table S2. Nominal significant association (*p*-value ≤ 0.05) between metabolites level in children at 6 months of age and in mothers at week 24 of pregnancy and at week 1 after birth and gross-motor milestone achievement.

			Child 6 Months		Mother Week 24		Mother Week 1	
**Super Pathway**	**Sub Pathway**	**Metabolite**	**β-estimate**	**p**	**β-estimate**	**p**	**β-estimate**	**p**
Amino Acid	Tyrosine Metabolism	**tyramine O-sulfate**	0.3001	0.0002(*)	−0.0184	0.8021	−0.0947	0.2032
		3-(4-hydroxyphenyl)lactate (HPLA)	−0.2769	0.0008	−0.0606	0.4147	−0.0431	0.5688
		thyroxine	−0.2069	0.0122	0.0995	0.1955	0.0561	0.4604
	Tryptophan Metabolism	indole-3-carboxylic acid	−0.1767	0.0358	Not Present	Not Present	Not Present	Not Present
		C-glycosyltryptophan	−0.1678	0.0418	−0.0024	0.9760	−0.0597	0.4434
		5-bromotryptophan	0.1750	0.0369	NA	NA	NA	NA
	Urea cycle; Arginine and Proline Metabolism	homocitrulline	−0.1930	0.0204	0.0947	0.2260	0.0037	0.9620
		N-methylproline	−0.1836	0.0250	0.0064	0.9324	−0.0599	0.4400
	Polyamine Metabolism	acisoga	−0.2245	0.0066	Not Present	Not Present	Not Present	Not Present
		(N(1) + N(8))-acetylspermidine	−0.1946	0.0182	−0.1300	0.0918	−0.0747	0.3199
	Methionine, Cysteine, SAM and Taurine Metabolism	N-formylmethionine	−0.2404	0.0033	−0.1530	0.0434	−0.2151	0.0049
		2,3-dihydroxy-5-methylthio-4-pentenoate (DMTPA)*	−0.1789	0.0344	−0.1140	0.1412	−0.0035	0.9635
	Lysine Metabolism	5-hydroxylysine	−0.2368	0.0041	−0.0684	0.3900	−0.2149	0.0047

			Child 6 Months		Mother Week 24		Mother Week 1	
**Super Pathway**	**Sub Pathway**	**Metabolite**	**β-estimate**	**p**	**β-estimate**	**p**	**β-estimate**	**p**
		N6,N6,N6-trimethyllysine	−0.1711	0.0372	−0.1083	0.1419	−0.0435	0.5615
	Leucine, Isoleucine and Valine Metabolism	3-methylglutarylcarnitine (2)	−0.1743	0.0327	−0.0379	0.6274	−0.0162	0.8311
	Histidine Metabolism	imidazole lactate	−0.2602	0.0020	−0.0613	0.4231	−0.0757	0.3124
		N-acetylcarnosine	−0.1771	0.0310	0.0117	0.8769	−0.0213	0.7875
		1-methyl-5-imidazoleacetate	−0.1907	0.0211	0.0343	0.6566	−0.0057	0.9448
	Glycine, Serine and Threonine Metabolism	threonine	−0.1919	0.0227	-0.2358	0.0022	−0.1448	0.0544
		N-acetylthreonine	−0.1771	0.0337	−0.0340	0.6496	−0.1381	0.0699
	Glutathione Metabolism	cysteinylglycine disulfide*	−0.1664	0.0424	0.0376	0.6409	−0.0519	0.5080
		cys-gly, oxidized	−0.1646	0.0449	0.0767	0.3438	0.0302	0.6951
	Glutamate Metabolism	pyroglutamine*	−0.2191	0.0085	0.0002	0.9974	−0.0779	0.3314
	Creatine Metabolism	creatinine	−0.2377	0.0038	−0.0699	0.3576	−0.0794	0.2980
		creatine	0.2036	0.0157	−0.1063	0.1613	0.0381	0.6173
	Alanine and Aspartate Metabolism	N-acetylalanine	−0.2516	0.0020	−0.0218	0.7750	−0.0468	0.5381
		hydroxyasparagine	−0.2228	0.0078	−0.0830	0.3096	−0.0951	0.2376
Carbohydrate	Fructose, Mannose and Galactose Metabolism	fructose	−0.2237	0.0061	−0.0495	0.5336	−0.0963	0.1951
		mannitol/sorbitol	0.2023	0.0142	−0.0242	0.7594	−0.0890	0.2560
		mannose	−0.1685	0.0401	0.0638	0.4246	0.0128	0.8666

			Child 6 Months		Mother Week 24		Mother Week 1	
Super Pathway	Sub Pathway	Metabolite	β-estimate	p	β-estimate	p	β-estimate	p
Cofactors and Vitamins	Nicotinate and Nicotinamide Metabolism	trigonelline (N'-methylnicotinate)	−0.2147	0.0098	−0.0391	0.6406	0.0131	0.8733
Lipid	Sterol	7-HOCA	−0.1676	0.0424	0.0557	0.4778	−0.1138	0.1354
	Sphingolipid Metabolism	sphinganine-1-phosphate	−0.1870	0.0225	0.1424	0.0616	0.0667	0.3882
		sphingosine 1-phosphate	−0.1734	0.0352	0.1127	0.1423	0.0927	0.2324
	Primary Bile Acid Metabolism	glycochenodeoxycholate glucuronide (1)	0.1719	0.0374	0.0878	0.2662	0.0802	0.3484
		glycochenodeoxycholate	0.1689	0.0420	0.0221	0.7658	−0.0191	0.7965
	Plasmalogen	1-(1-enyl-palmitoyl)-2-arachidonoyl-GPC (P-16:0/20:4)*	−0.2312	0.0049	-0.0138	0.8580	−0.0882	0.2939
		1-(1-enyl-palmitoyl)-2-arachidonoyl-GPE (P-16:0/20:4)*	−0.1911	0.0202	0.0395	0.6073	−0.0384	0.6292
	Glycerolipid Metabolism	glycerophosphoglycerol	−0.1641	0.0487	0.0702	0.3601	0.0026	0.9713
	Fatty Acid Monohydroxy	2-hydroxydecanoate	−0.2605	0.0017	0.0889	0.2447	0.2050	0.0065
		5-hydroxyhexanoate	−0.1623	0.0494	Not Present	Not Present	Not Present	Not Present

			Child 6 Months		Mother Week 24		Mother Week 1	
**Super Pathway**	**Sub Pathway**	**Metabolite**	**β-estimate**	**p**	**β-estimate**	**p**	**β-estimate**	**p**
		3-hydroxyoleate*	−0.1758	0.0337	Not Present	Not Present	Not Present	Not Present
	Fatty Acid Amino	N-acetyl-2-aminooctanoate*	−0.1843	0.0311	0.0131	0.8602	−0.0845	0.2663
	Fatty Acid Metabolism (Acyl Carnitine)	octadecanedioylcarnitine (C18-DC)*	0.1804	0.0316	−0.0151	0.8430	−0.0518	0.5079
		linoleoylcarnitine (C18:2)*	−0.1724	0.0386	0.1135	0.1544	−0.0330	0.6711
		arachidonoylcarnitine (C20:4)	−0.1643	0.0477	−0.0291	0.7096	−0.0048	0.9520
Unknown	Unknown	X - 12216	0.1920	0.0193	0.0809	0.3229	0.0338	0.7064
		X - 18913	−0.1902	0.0218	0.1107	0.1481	0.1804	0.0194
		X - 11795	0.1839	0.0277	−0.0046	0.9511	−0.0500	0.5081
		X - 17185	0.1746	0.0320	0.0697	0.3709	0.0503	0.5707
		X - 12026	−0.1831	0.0279	0.0846	0.2748	0.0163	0.8383
		X - 23680	−0.1738	0.0488	−0.0045	0.9564	−0.0015	0.9873
		X - 17335	−0.1774	0.0402	Not Present	Not Present	Not Present	Not Present
Nucleotide	Pyrimidine Metabolism, Uracil containing	pseudouridine	−0.1803	0.0285	−0.0868	0.2532	−0.1278	0.1026
		uridine	−0.1685	0.0401	0.0171	0.8197	0.0033	0.9652

			Child 6 Months		Mother Week 24		Mother Week 1	
**Super Pathway**	**Sub Pathway**	**Metabolite**	**β-estimate**	**p**	**β-estimate**	**p**	**β-estimate**	**p**
	Pyrimidine Metabolism, Cytidine containing	2'-O-methylcytidine	−0.1925	0.0196	0.0769	0.3098	0.0657	0.3820
	Purine Metabolism, Adenine containing	N6-carbamoylthreonyladenosine	−0.1966	0.0170	−0.0987	0.1981	−0.1664	0.0305
	Purine Metabolism, (Hypo)Xanthine/Inosine containing	N1-methylinosine	−0.2242	0.0063	−0.0457	0.5738	−0.0978	0.2135
		urate	−0.1621	0.0477	−0.0775	0.3209	−0.1025	0.1858
Peptide	Gamma-glutamyl Amino Acid	gamma-glutamylhistidine	−0.1853	0.0236	−0.1526	0.0488	−0.1965	0.0090
Xenobiotics	Food Component/Plant	stachydrine	−0.1967	0.0173	−0.0009	0.9906	−0.0489	0.5300
		(S)-a-amino-omega-caprolactam	−0.2555	0.0019	−0.0028	0.9698	−0.0171	0.8357

(*): FDR ≤ 0.25

The metabolites highlighted in **bold** and *italic* are replicated in the mother's metabolome.

Figure S1. Correlation map (based on Spearman correlation) of the nominal significant metabolites in the association between child 6mo metabolome and gross motor milestone.

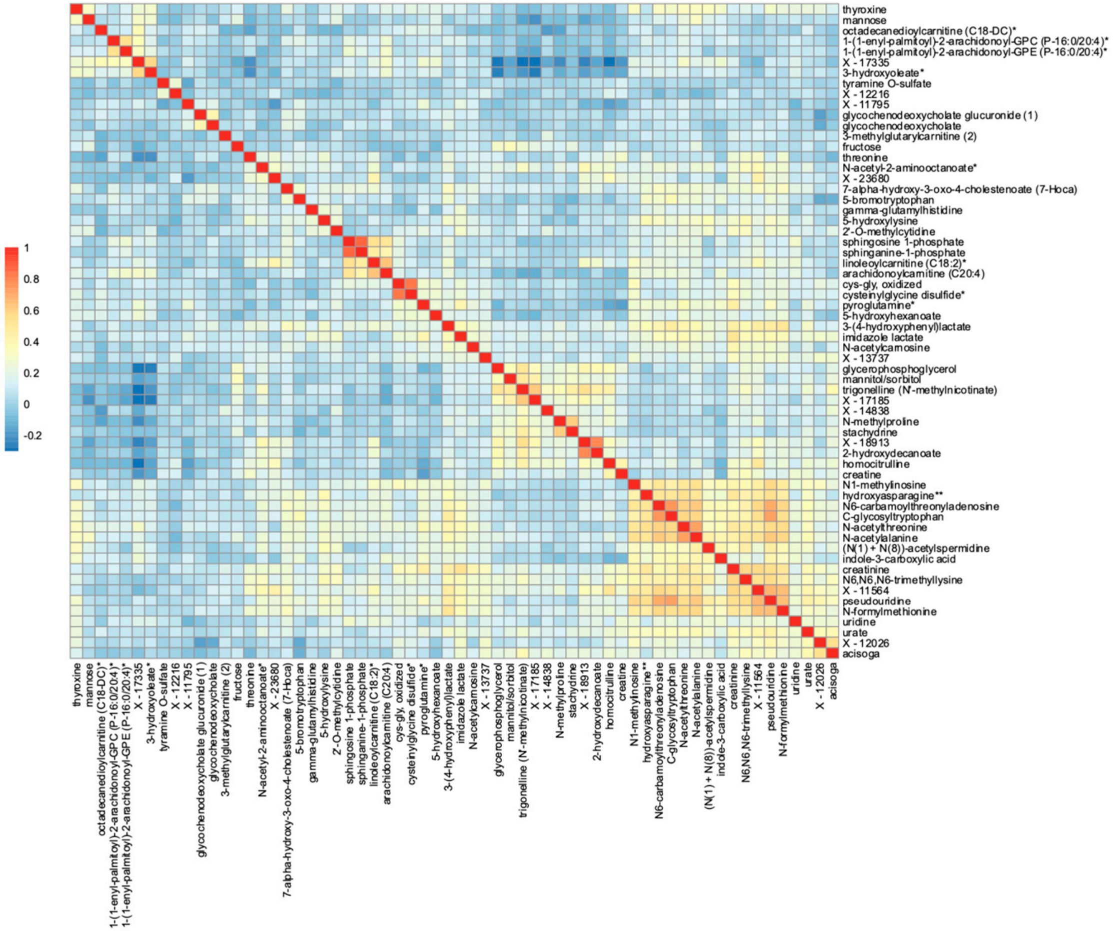

Figure S2. Correlation map (based on Spearman correlation) between the significant metabolite level in the child at 6mo and the mother at w24 and w1.

Mw1: Mother at week1; Mw24: Mother at week 24.

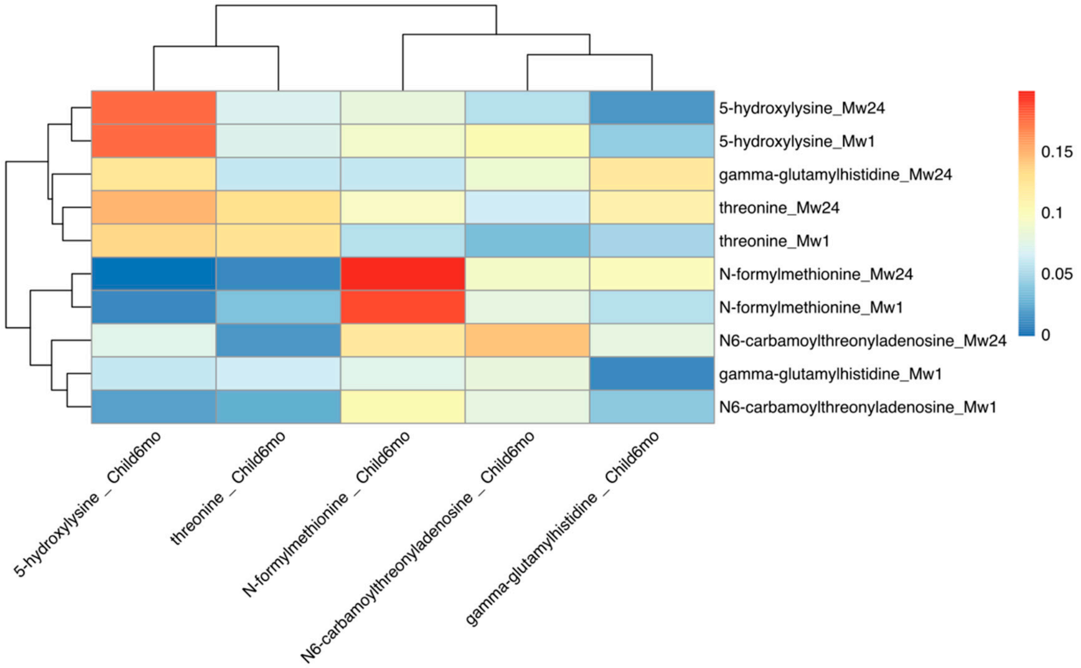

Table S3. Association between metabolites level in children at 6 months of age and gross-motor milestone achievement. Analysis adjusted for mother's metabolite level at w24 and w1.

Super Pathway	Sub Pathway	Metabolite	Mother´s Level Adjustment	β-estimate	p	CI Low	CI High
Amino Acid	Tyrosine Metabolism	tyramine O-sulfate	tyramine O-sulfate_w24	0.3010	0.0002	0.1419	0.4601
		tyramine O-sulfate	tyramine O-sulfate_w1	0.3060	0.0002	0.1460	0.4660
	Glycine, Serine and Threonine Metabolism	Threonine	Threonine_w24	−0.1876	0.0289	−0.3557	−0.0194
		Threonine	Threonine_w1	−0.1865	0.0311	−0.3560	−0.0170
	Lysine Metabolism	5-hydroxylysine	5-hydroxylysine_w24	−0.2248	0.0074	−0.3891	−0.0605
		5-hydroxylysine	5-hydroxylysine_w1	−0.2050	0.0144	−0.3690	−0.0411
	Methionine, Cysteine, SAM and Taurine Metabolism	N-formylmethionine	N-formylmethionine_w24	−0.2030	0.0168	−0.3693	−0.0367
		N-formylmethionine	N-formylmethionine_w1	−0.2132	0.0120	−0.3793	−0.0471
Nucleotide	Purine Metabolism, Adenine containing	N6-carbamoylthreonyladenosine	N6-carbamoylthreonyladenosine_w24	−0.1829	0.0318	−0.3499	−0.0160
		N6-carbamoylthreonyladenosine	N6-carbamoylthreonyladenosine_w1	−0.1897	0.0213	−0.3510	−0.0284
Peptide	Gamma-glutamyl Amino Acid	gamma-glutamyl histidine	gamma-glutamyl histidine_w24	−0.1782	0.0324	−0.3414	−0.0150
		gamma-glutamyl histidine	gamma-glutamyl histidine_w1	−0.1873	0.0225	−0.3480	−0.0265

Table S4. Metabolites in the PLS-DA model and their associated VIP value.

Metabolite	Sub Pathway	VIP
hydroxyasparagine**	Alanine and Aspartate Metabolism	0.817
N-acetylalanine		0.965
oxalate (ethanedioate)	Ascorbate and Aldarate Metabolism	0.830
Creatine	Creatine Metabolism	***1.471***
Isoleucylglycine	Dipeptide	0.911
Valylglycine		0.960
N-linoleoyltaurine*	Endocannabinoid	0.706
butyrylcarnitine (C4)	Acyl Carnitine	0.868
octadecanedioylcarnitine (C18-DC)*		0.951
3-methylglutarylcarnitine (2)		1.012
octanoylcarnitine (C8)		1.002
N-acetyl-2-aminooctanoate*	Fatty Acid	1.025
2-hydroxyglutarate		1.075
12,13-DiHOME		0.998
9,10-DiHOME		1.016
13-HODE + 9-HODE		1.036
2-hydroxydecanoate		***1.201***
2-hydroxyoctanoate		0.977
9-hydroxystearate		0.947
Ergothioneine	Food Component	0.876
Stachydrine		1.030
(S)-a-amino-omega-caprolactam		0.922
ethyl alpha-glucopyranoside		0.862
Fructose	Fructose, Mannose and Galactose Metabolism	1.090
gamma-glutamylhistidine	Gamma-glutamyl Amino Acid	0.857
Glutamate	Glutamate Metabolism	1.034
pyroglutamine*		0.892
imidazole lactate	Histidine Metabolism	***1.171***
imidazole propionate		0.933
1-methyl-5-imidazoleacetate		0.931
Isoleucine	Leucine, Isoleucine and Valine Metabolism	0.986
5-hydroxylysine	Lysine Metabolism	***1.179***
Lysine		0.942
hydroxy-N6, N6, N6-trimethyllysine		0.877
N-formylmethionine	Methionine, Cysteine, SAM and Taurine Metabolism	0.931
1-linoleoylglycerol (18:2)	Monoacylglycerol	0.924
X – 12026	Unknown	0.898

Metabolite	Sub Pathway	VIP
X – 12216		1.055
X – 12456		***1.254***
X – 17185		0.830
X – 17676		***1.245***
X – 18913		1.109
X – 21286		1.015
X – 21628		0.964
X – 24571		***1.222***
X – 24699		0.891
trigonelline (N'-methylnicotinate)	Nicotinate and Nicotinamide Metabolism	1.093
glucuronide of C12H22O4 (1)*	Partially Characterized Molecules	0.973
1-stearoyl-2-docosahexaenoyl-GPE (18:0/22:6)*	Phosphatidylethanolamine (PE)	1.022
1-(1-enyl-palmitoyl)-2-arachidonoyl-GPC (P-16:0/20:4)*	Plasmalogen	0.854
Glycochenodeoxycholate	***Primary Bile Acid Metabolism***	***1.189***
N1-methylinosine	Purine Metabolism, (Hypo)Xanthine/Inosine containing	1.181
N4-acetylcytidine	Pyrimidine Metabolism, Cytidine containing	0.937
sphinganine-1-phosphate	Sphingolipid Metabolism	1.013
3-indoxyl sulfate	Tryptophan Metabolism	0.872
5-bromotryptophan		1.051
3-(4-hydroxyphenyl)lactate	***Tyrosine Metabolism***	0.928
3-methoxytyramine sulfate		0.732
3-methoxytyrosine		0.918
Thyroxine		0.813
tyramine O-sulfate		***1.119***
N-methylproline	Urea cycle; Arginine and Proline Metabolism	1.048

The metabolites in **bold and *italic*** are the most important in the PLS-DA model

Figure S3. Results from the PLS-DA model validation. **A**: The histogram represents the distribution of the averaged values (from 100 test-sets) of AUC results from the permutation test; **B**: The histogram represents the distribution of the averaged values (from 100 test-sets) of Classification Error (CE) results from the permutation test. The red vertical lines represent the averaged value (from 100 test set) from the original class.

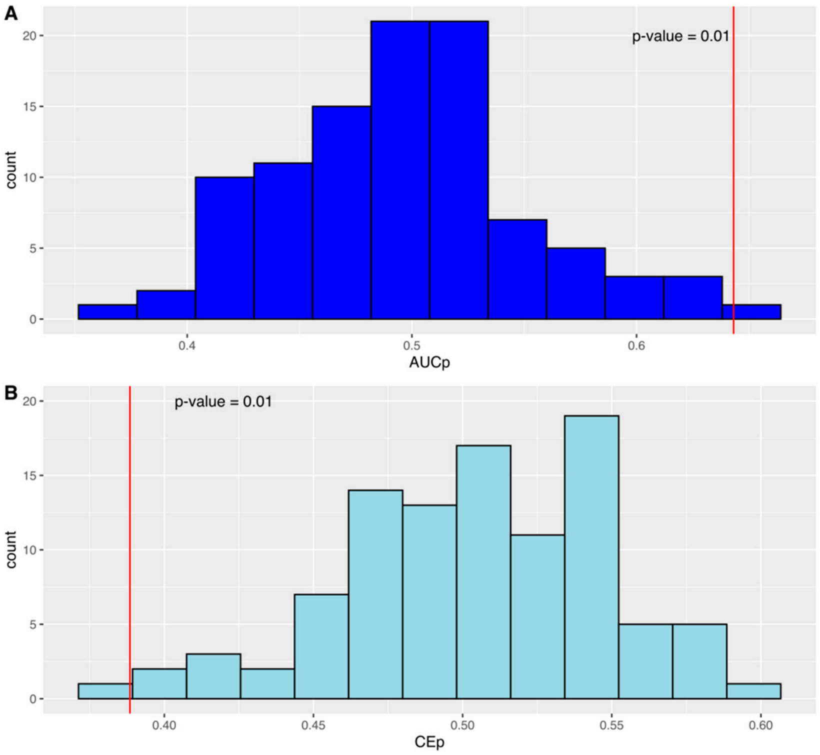

Table S5. Nominal significant association (*p*-value ≤ 0.05) between metabolites level in children at 6 months of age and early motor milestones.

Super Pathway	Sub Pathway	Metabolite	β-estimate	p	CI Low	CI High
Amino Acid	Alanine and Aspartate Metabolism	N-acetylalanine	0.1863	0.0036	0.0612	0.3114
		hydroxyasparagine**	0.1489	0.0237	0.0199	0.2778
	Creatine Metabolism	creatine	−0.1744	0.0080	−0.3029	−0.0458
	Glutamate Metabolism	pyroglutamine*	0.1516	0.0206	0.0234	0.2798
		N-acetyl-aspartyl-glutamate (NAAG)	0.1423	0.0267	0.0165	0.2681
	Glutathione Metabolism	5-oxoproline	0.1734	0.0070	0.0476	0.2992
	Glycine, Serine and Threonine Metabolism	N-acetylthreonine	0.2042	0.0018	0.0767	0.3317
		N-acetylserine	0.1603	0.0136	0.0331	0.2875
	Histidine Metabolism	carnosine	0.1680	0.0100	0.0403	0.2957
		1-methylhistidine	0.1541	0.0171	0.0276	0.2807
	Leucine, Isoleucine and Valine Metabolism	N-acetylvaline	0.1408	0.0283	0.0150	0.2665
	Lysine Metabolism	5-hydroxylysine	0.1754	0.0067	0.0489	0.3020
		5-(galactosylhydroxy)-L-lysine	0.1430	0.0262	0.0170	0.2690
		N6,N6,N6-trimethyllysine	0.1414	0.0280	0.0154	0.2675
	Methionine, Cysteine, SAM and Taurine Metabolism	N-formylmethionine	0.2217	0.0006	0.0963	0.3471
		5-methylthioribose**	0.1385	0.0311	0.0126	0.2644
		methionine sulfone	0.1323	0.0418	0.0049	0.2596
		2,3-dihydroxy-5-methylthio-4-pentenoate (DMTPA)*	0.1825	0.0060	0.0527	0.3123
	Polyamine Metabolism	N1,N12-diacetylspermine	0.1946	0.0026	0.0684	0.3207
		acisoga	0.1552	0.0165	0.0284	0.2820
		4-acetamidobutanoate	0.1435	0.0258	0.0174	0.2695
		N-acetylputrescine	0.1436	0.0266	0.0168	0.2705
		N-acetyl-isoputreanine*	0.1286	0.0457	0.0024	0.2547
	Tryptophan Metabolism	indolelactate	0.1521	0.0266	0.0177	0.2866
		N-acetyltryptophan	0.1400	0.0306	0.0131	0.2668
	Tyrosine Metabolism	vanillactate	0.1816	0.0046	0.0562	0.3070
		tyrosine	−0.1619	0.0137	−0.2905	−0.0333

Super Pathway	Sub Pathway	Metabolite	β-estimate	p	CI Low	CI High
		3-(4-hydroxyphenyl)lactate	0.1414	0.0294	0.0142	0.2685
		N-acetyltyrosine	0.1335	0.0382	0.0073	0.2596
	Urea cycle; Arginine and Proline Metabolism	dimethylarginine (SDMA + ADMA)	0.1568	0.0157	0.0298	0.2839
Carbohydrate	Pentose Metabolism	xylose	0.1338	0.0375	0.0078	0.2597
Energy	TCA Cycle	succinate	0.1630	0.0127	0.0349	0.2911
		malate	0.1537	0.0166	0.0280	0.2794
		succinylcarnitine (C4-DC)	0.1439	0.0255	0.0177	0.2700
		aconitate [cis or trans]	0.1422	0.0281	0.0153	0.2691
Lipid	Androgenic Steroids	andro steroid monosulfate C19H28O6S (1)*	0.1426	0.0312	0.0129	0.2723
	Ceramides	glycosyl ceramide (d18:2/24:1, d18:1/24:2)*	0.1452	0.0342	0.0109	0.2796
	Fatty Acid Metabolism(Acyl Carnitine)	dihomo-linoleoylcarnitine (C20:2)*	0.1883	0.0040	0.0605	0.3161
		oleoylcarnitine (C18:1)	0.1551	0.0166	0.0283	0.2818
		linoleoylcarnitine (C18:2)*	0.1509	0.0209	0.0230	0.2789
		adipoylcarnitine (C6-DC)	0.1534	0.0209	0.0234	0.2834
		cerotoylcarnitine (C26)*	−0.1489	0.0220	−0.2762	−0.0215
		arachidonoylcarnitine (C20:4)	0.1463	0.0242	0.0192	0.2735
		docosapentaenoylcarnitine (C22:5n3)*	0.1493	0.0272	0.0169	0.2818
		pimeloylcarnitine/3-methyladipoylcarnitine (C7-DC)	0.1435	0.0300	0.0139	0.2731
	Fatty Acid, Monohydroxy	2-hydroxydecanoate	0.1763	0.0066	0.0492	0.3034
	Lysophospholipid	1-linolenoyl-GPC (18:3)*	−0.1708	0.0087	−0.2983	−0.0434
	Polyunsaturated Fatty Acid (n3 and n6)	hexadecatrienoate (16:3n3)	−0.1390	0.0330	−0.2667	−0.0113
	Pregnenolone Steroids	21-hydroxypregnenolone monosulfate (2)	0.1444	0.0318	0.0126	0.2762
	Primary Bile Acid Metabolism	glycocholate	0.1887	0.0038	0.0611	0.3163
		taurocholate	0.1723	0.0075	0.0462	0.2983

Super Pathway	Sub Pathway	Metabolite	β-estimate	p	CI Low	CI High
	Secondary Bile Acid Metabolism	taurohyocholate*	0.1780	0.0059	0.0516	0.3045
		glycohyocholate	0.1655	0.0117	0.0369	0.2940
	Sphingolipid Metabolism	sphingomyelin (d17:1/16:0, d18:1/15:0, d16:1/17:0)*	−0.1743	0.0072	−0.3012	−0.0473
		sphingosine 1-phosphate	0.1553	0.0162	0.0289	0.2818
		sphingomyelin (d18:1/21:0, d17:1/22:0, d16:1/23:0)*	−0.1529	0.0319	−0.2925	−0.0133
	Sterol	7-alpha-hydroxy-3-oxo-4-cholestenoate (7-Hoca)	0.1784	0.0056	0.0525	0.3043
Unknown	Unknown	X - 24293	−0.2051	0.0017	−0.3326	−0.0775
		X - 24952	−0.1983	0.0019	−0.3229	−0.0736
		X - 23739	0.1831	0.0054	0.0543	0.3119
		X - 11564	0.1825	0.0060	0.0527	0.3123
		X - 12026	0.1790	0.0060	0.0515	0.3064
		X - 09789	0.1734	0.0075	0.0466	0.3002
		X - 12407	−0.1748	0.0096	−0.3069	−0.0427
		X - 24545	0.1684	0.0129	0.0359	0.3009
		X - 23666	0.1525	0.0173	0.0271	0.2780
		X - 13737	0.1512	0.0194	0.0245	0.2778
		X - 16938	0.1497	0.0211	0.0226	0.2768
		X - 24307	0.1469	0.0238	0.0196	0.2742
		X - 16397	0.1462	0.0275	0.0163	0.2762
		X - 18913	0.1383	0.0327	0.0114	0.2653
		X - 12906	0.1392	0.0337	0.0108	0.2676
		X - 16580	0.1439	0.0345	0.0106	0.2772
		X - 18887	0.1320	0.0413	0.0053	0.2587
		X - 24544	0.1319	0.0428	0.0043	0.2594
		X - 23196	0.1317	0.0435	0.0039	0.2596
Nucleotide	Purine Metabolism, (Hypo)Xanthine/Inosine containing	N1-methylinosine	0.1815	0.0048	0.0556	0.3074
		urate	0.1716	0.0074	0.0462	0.2969
	Purine Metabolism, Adenine containing	N6-carbamoylthreonyladenosine	0.2320	0.0003	0.1065	0.3575

Super Pathway	Sub Pathway	Metabolite	β-estimate	p	CI Low	CI High
	Purine Metabolism, Guanine containing	N2,N2-dimethylguanosine	0.1583	0.0138	0.0324	0.2842
	Pyrimidine Metabolism, Orotate containing	dihydroorotate	0.1907	0.0033	0.0638	0.3176
	Pyrimidine Metabolism, Thymine containing	5,6-dihydrothymine	0.1774	0.0059	0.0514	0.3035
	Pyrimidine Metabolism, Uracil containing	pseudouridine	0.1686	0.0090	0.0422	0.2949
		uridine	0.1264	0.0496	0.0002	0.2526
Partially Characterized Molecules	Partially Characterized Molecules	glucuronide of C12H22O4 (1)*	0.1406	0.0315	0.0125	0.2687
Peptide	Gamma-glutamyl Amino Acid	gamma-glutamylglutamine	0.1322	0.0419	0.0048	0.2596
		gamma-glutamyltryptophan	0.1307	0.0420	0.0047	0.2566
Xenobiotics	Chemical	succinimide	0.1438	0.0275	0.0160	0.2716
	Food Component/Plant	tartarate	−0.1405	0.0310	−0.2682	−0.0129
	Xanthine Metabolism	7-methylxanthine	−0.1475	0.0231	−0.2746	−0.0204

Figure S4. Biplot from principal component analysis of all the 13 milestones. A: Biplot from principal component analysis of all the 13 milestones. Principal component 1 and 2 (PC1 and PC2) explain 51% and 21 % of the overall variation in the data, respectively. B: Biplot from principal component analysis of the 7 early motor milestones. Principal component 1 and 2 (PC1 and PC2) explain 45% and 17 % of the overall variation in the data, respectively. C: Biplot from principal component analysis of the 5 gross motor milestones. Principal component 1 and 2 (PC1 and PC2) explain 50 % and 23 % of the overall variation in the data, respectively.

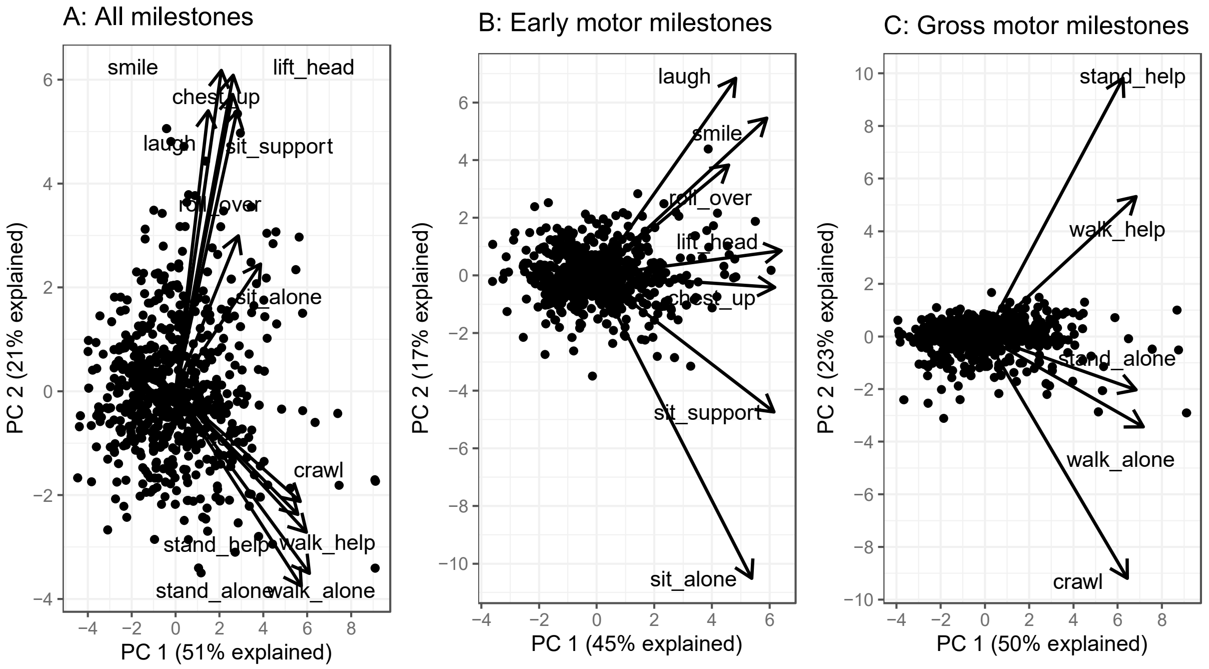

Supplement: Supplementary file 1 [file metabolites-10-00337-s001.pdf]
